# Incidence, predictors and outcome of neonatal-onset intestinal failure

**DOI:** 10.1016/j.intf.2025.100333

**Published:** 2025-11-28

**Authors:** Rishi Bolia, Shay McLaren, Sara Alremawi, Looi C. Ee

**Affiliations:** Department of Gastroenterology, Hepatology and Liver Transplant, Queensland Children’s Hospital, Brisbane, Australia

**Keywords:** Intestinal failure, Enteral autonomy, Short bowel syndrome, Parenteral nutrition

## Abstract

**Background:**

Neonatal-onset Intestinal Failure (IF) and Short Bowel Syndrome (SBS) are associated with significant morbidity. We aimed to estimate incidence, identify predictors and determine outcomes of neonatal IF and SBS.

**Materials and methods:**

Retrospective review of children admitted to Queensland Children’s Hospital between April, 2018 to March, 2022 who received parenteral nutrition (PN) in the neonatal period. IF was defined as those who required PN> 60 days from a gastrointestinal cause. Population-based estimate of incidence was calculated using census data from the national bureau of statistics.

**Results:**

49 neonatal patients received PN, of which 21 had IF, including 16 with SBS. The population-based incidence of IF and SBS-IF were 13.7(95 % CI 10.0–17.1) and 10.51(95 % CI 5.04–15.59) per 100,000 live-births respectively. Those with IF had lower birth weight [1985(IQR 1321)g vs.3140(IQR778)g, p = 0.001], lower gestational age [34weeks(IQR7.5)vs.38weeks(IQR3),p = 0.001], shorter residual small bowel [45 cm(IQR44)vs.245 cm(IQR50),p = 0.001], more likely to have an enterostomy [17/21vs.10/28,p = 0.003] and no colon [10/21vs.3/28,p = 0.0001] compared to those without IF. On multivariate analysis, residual small bowel length [OR 0.73(95 %CI 0.57–0.93),p = 0.008] and absent colon [OR 0.84(95 %CI 0.69 – 0.92),p = 0.001] were independent predictors of IF. At follow-up, 71 % (15/21) of patients with IF, including 81 % (13/16) with SBS attained enteral autonomy with no mortality. Children who attained enteral autonomy had significantly longer residual bowel [47.5(IQR 33)cm. vs. 26 cm.,p = 0.04] as compared to those who remained on PN.

**Conclusion:**

The population-based incidence of IF and SBS-IF in Queensland, Australia was 13.7 and 10.51 per 100,000 live-births respectively. Most children with neonatal IF achieve enteral autonomy with residual small bowel length predictive of it.

## Introduction

Intestinal Failure (IF) is a heterogeneous condition characterized by the inability of the patient’s intestine to absorb required fluids and/or nutrients for adequate growth and homeostasis. The most prevalent conditions in children leading to IF include short bowel syndrome, gastrointestinal motility disorders and congenital enteropathies [1].

Advancements in neonatal intensive care, parenteral nutrition and surgical strategies have improved the survival of children with IF [2]. Despite these advances, IF continues to be associated with significant morbidity. An accurate estimate of the prevalence of this problem would be useful to enable better allocation of resources and further improve care. There is however, a paucity of information in the literature on the incidence of IF. Until recently, there was no consensus on the definition of IF, with some based on residual bowel length while others on varying duration of parenteral nutrition support.

The management of IF is multi-disciplinary, with attainment of enteral autonomy being the main aim. Several factors are recognised to influence outcomes including gestation and age at surgery, location and extent of bowel loss, presence of ileocecal valve, colonic continuity and the mode and type of enteral feeding. Variability in institutional practices and management strategies have led to differences in rates of enteral autonomy between centres [3].

Children with IF despite weaning off PN remain at risk of undernutrition and growth abnormalities. There is very little data about the follow–up and anthropometric outcomes of these children [4]. We aimed to estimate the incidence of intestinal failure in our population and to identify the predictors and cumulative incidence of enteral autonomy. We also aimed to evaluate the anthropometric outcomes of children with IF who achieved enteral autonomy.

## Methods

Queensland Children’s Hospital (QCH) is the main tertiary pediatric hospital in Queensland, Australia and has the only dedicated intestinal rehabilitation and intestinal failure service for the state. Retrospective review of electronic records was used to identify all children admitted to QCH between April, 2018 and March, 2022 who received parenteral nutrition (PN) in the neonatal period for a gastrointestinal cause. All children were born in or after 2018. From this cohort, children with IF were identified. IF was defined as critical reduction of the gut mass or function necessitating PN for adequate growth for minimum 60 days within a 74 consecutive day interval [1]. Children in whom IF was a consequence of gut resection were labelled SBS-IF.

Data regarding gender, birth weight, gestational age, underlying diagnosis, surgical and post-operative details including residual small bowel length (if applicable), duration of PN, complications and outcomes at last follow-up (on home PN/enteral autonomy/mortality) were recorded. The residual small intestinal length was also calculated as percentage of expected bowel length normalized for postconception age [5]. IFALD was defined according to the 2015 ESPGHAN paper: bilirubin> 100 μmol/L > 2–4 weeks [6]. Central-line associated bloodstream infection (CLABSI) was defined as a laboratory-confirmed bloodstream infection in a patient where the central line was in place for > 48 h on the date of the event and the organism cultured from blood is not related to an infection at another site [7]. All children were followed until 31st March, 2024 to ensure a minimum 2 year follow-up period for all patients. Anthropometric parameters at 1 and 2 years of age were recorded.

The outcome variable for the study was the achievement of enteral autonomy, defined as discontinuation of PN for > 3 consecutive months with maintenance of acceptable growth parameters [1].

The population-based incidence of intestinal failure (IF) and short bowel syndrome–associated intestinal failure (SBS-IF) was calculated as the number of new cases per year divided by the total number of live births in Queensland during the same period. Incidence was expressed per 100,000 live births.

New cases were defined as all children admitted to Queensland Children’s Hospital (QCH) for intestinal rehabilitation during the study period, received parenteral nutrition (PN) initiated in the neonatal period and met diagnostic criteria for IF or SBS-IF. Although QCH does not have an on-site neonatal intensive care unit (NICU), all four tertiary NICUs in Queensland—Mater Mother’s Hospital, Royal Brisbane and Women’s Hospital, Gold Coast University Hospital, and Townsville University Hospital—refer infants requiring prolonged PN for a gastrointestinal indication to QCH for intestinal rehabilitation. To minimize case ascertainment bias, central pharmacies at each referral hospital were contacted to verify whether PN had been dispensed for a gastrointestinal indication more than 60 consecutive days to any infant during the study period.

Data on live births were obtained from the Australian Bureau of Statistics (ABS). Our study period included data from the years 2018 – 2022. However, as the entire year in 2018 and 2022 was not included and only annual live – birth data for the state was available on the ABS website we included only the years 2019–2021 for incidence calculation. Ninety-five percent confidence intervals were included with each estimate.

## Statistical methods

All data were represented as median (Q1-Q3) or percentages (%). Mann-Whitney’s test was used to compare unpaired continuous variables and Fisher’s exact test for dichotomous variables. A p value < 0.05 was considered significant. Statistical analyses were performed using the IBM Statistical Package for the Social Sciences v.26.0 (SPSS, Armonk, NY; IBM Corp).

The study was approved by the Queensland Children's Hospital Human Research Ethics

## Results

### Baseline characteristics

**Fig. 1:**
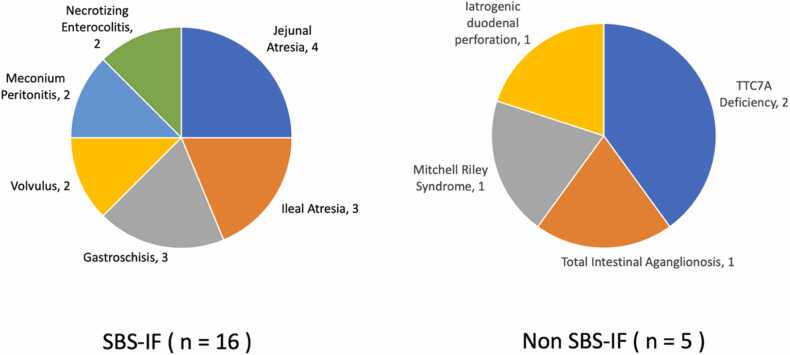
Etiology of Short Bowel Syndrome.

Forty-nine patients were noted to have received PN in the neonatal period, of which 21 children had IF including 16 with SBS-IF. The causes of SBS in those with SBS-IF included jejunal and/or ileal atresia, gastroschisis, necrotizing enterocolitis, volvulus and meconium peritonitis (Fig. 1). The etiologies of IF in the 5 children without SBS included TTC7A deficiency, Mitchell-Riley syndrome, total intestinal aganglionosis and iatrogenic duodenal perforation.

### Incidence

The population-based incidence of IF and SBS-IF were calculated to be 13.7 (95 % CI 10.0–17.1) and 10.51 (95 % CI 5.04–15.59) per 100,000 live-births respectively.

### Predictors of IF (Table 1)

Children with IF had lower birth weight, younger gestational age at birth, shorter length of residual small bowel, were more likely to have an enterostomy, and absent colon when compared to those without IF. On multivariate analysis, residual small bowel length [OR 0.73 (95 %CI 0.57–0.93), p = 0.008] and absent colon [OR 0.84 (95 %CI 0.69–0.92), p = 0.001] were independent predictors of developing IF.

**Table 1:** Predictors of Intestinal Failure.

	**IF (n = 21)**	**Non IF (n = 28)**	**p value**
Gender (Males)	9	19	0.09
Birth weight (g)	1985 (1324.5–2645)	3140 (2751–3529)	0.001
Gestation (weeks)	34 (30.25–37.25)	38 (36.5–39.5)	0.001
Bowel resection	16 (76 %)	7 (25 %)	0.0005
Absent colon	10 (48 %)	3 (11 %)	0.0001
Residual small bowel (cm)	45 (23−67)	245 (220−270)	0.001
Stoma present*	17 (81 %)	10 (36 %)	0.003

* at initial bowel resection

### Outcome

At follow-up, 71 % (15/21) of neonates with IF, including 81 % of those with SBS-IF, achieved enteral autonomy at median 156 (67−709) days. There was no mortality. The remaining 6 children continued onto home PN in a median follow-up of 1460 (781–1910) days. The etiology of the 6 children discharged on home PN included 3 with SBS-IF, 2 with TTC7A and 1 with Waardenburg-Shah syndrome who had total intestinal aganglionosis. The following complications related to PN occurred during the study period – central line associated blood stream infections (6 episodes in 4 patients, 1.4 per 1000 Home parenteral nutrition days), damaged/ fractured central venous access disease (10 episodes in 5 patients) and intestinal-failure associated liver disease in 1 patient.

Among children with SBS-IF (n = 16), children who attained enteral autonomy (n = 13) had a significantly longer residual bowel length [47.5(IQR 33) cm vs. 26 cm, p = 0.04] as compared to those who remained on PN (Table 2).

**Table 2:** Predictors of Enteral Autonomy.

	**Enteral Autonomy** **(n = 13)**	**Home PN** **(n = 3)**	**p value**
Males	7	2	0.65
Birth weight (g)	1985 (1112–2858)	1895(1539–2251)	0.91
IUGR	5	1	0.63
Gestation (weeks)	35 (31–39.5)	34 (32.5–36.5)	0.91
Residual small bowel (cm)	47.5 (31.5–64)	26	0.04
Residual small bowel (<25 %)	2	3	0.04
Stoma present*	12	2	0.10
IC valve present	11	2	0.17
> 2 bowel surgeries	5	2	0.17

* at initial bowel resection

Six patients needed nasogastric/gastrostomy feeds (either exclusively/ partially for top-up feeds) for 1–36 months after cessation of PN. At last follow – up one patient continues on overnight gastrostomy feeds. Four patients had oral aversion.

At last follow-up, 81 % (17/21) had normal height and weight with Z scores > -2.0.

## Discussion

We estimated the population-based incidence of IF and SBS-IF in Queensland, Australia to be 13.7 and 10.51 per 100,000 live-births respectively. In perhaps the only other study in which a similar estimate was made, a Canadian Collaborative Study Group in 2004 estimated the neonatal incidence of SBS-IF as 24.5 per 100.000 live births, which is more than double our estimate [8]. These data sets however are not completely comparable, as SBS-IF was defined differently in the Canadian study. They defined SBS-IF as the need for PN > 42 days after bowel resection and/or residual small bowel length of less than 25 % expected for gestational age. Nevertheless, it is likely that the advances in surgical, nutritional, neonatal intensive care and intestinal rehabilitation practices have resulted in some children with SBS being weaned off PN before the 60 day threshold.

Over the last two decades, survival in children with IF has steadily increased. Wales et al. reported a mortality of 37.5 % in a single centre study comprising of 40 neonates with SBS-IF in 2005 [8]. A large multicentre study from 2012 of 272 patients from 14 centres across USA and Canada between 2000 and 2004 reported a mortality of 27 % [9]. A large multicentre consortium comprising of 443 patients reported a 10.5 % mortality [2]. We did not have any deaths during the study period. A recent single centre study also reported zero mortality [10]. It is possible however that some patients were palliated or died prior to receiving prolonged PN so were not captured in this retrospective review, but unlikely to have contributed to the dramatic reduction in mortality over time.

Together with improved survival, the rate of achievement of enteral autonomy has also improved with better intestinal rehabilitation practices. 71 % of our patients with IF, including 81 % with SBS achieved enteral autonomy. This is definitely towards the higher side on comparison with other centres which report EA rates of 43–81 % [2], [6], [11], [12], [13], [14], [15], [16], [17]. Improved intestinal rehabilitation practices are the most likely reason for this, which has also resulted in reduced referrals for intestinal transplantation in recent years [2], [3], [18]. Interestingly, EA is reported to be lower in intestinal transplant centers, but this likely reflects referral bias [2]. Teduglutide, a recombinant analog of the intestinal hormone glucagon-like peptide 2 is also a contributor to improved EA rates worldwide [19], [20]. None of our cohort achieved EA from teduglutide use, since it is only available in Australia from aged 2 years although 1 child on home PN has since been commenced on it.

We found that residual bowel length and an absent colon were independent predictors of development of IF. The colon can compensate for the loss of small bowel and in a large retrospective cohort study it was found that if > 50 % of the colon is intact then it can compensate for a < 50 % small bowel length with similar EA rates as those in children with residual SB > 50 % [21]. In our cohort, residual small bowel length was the only predictor of EA. This apparent lack of effect of the presence or absence of the colon in predicting EA in our cohort is because nearly all our cohort had intact colon.

In today’s era, improvements in delivering nutrition, regular monitoring and a multidisciplinary approach have improved the nutritional outcomes of children with IF. All our patients fared well from the growth perspective with both height and weight within the normal range. Roggero et al. in their study from Milan demonstrated similar results with all infants showing similar growth patterns irrespective of the fact whether they were weaned off PN or not [13].

The strength of our study is the fact that it provides an updated incidence of neonatal-onset IF, something that had not been re-visited since 2004. All patients were managed by the same clinical team, avoiding any variations in clinical practise. There was no missing data. Limitations include the retrospective nature and small sample size of this study.

To conclude, neonates with IF have a good prognosis with 100 % survival and 70 % achieving enteral autonomy.

## CRediT authorship contribution statement

**Ee Looi C:** Writing – review & editing, Supervision, Methodology, Conceptualization. **Sara Alremawi:** Writing – review & editing, Data curation. **Shay McLaren:** Writing – review & editing, Methodology, Data curation, Conceptualization. **Rishi Bolia:** Writing – review & editing, Writing – original draft, Methodology, Formal analysis, Data curation, Conceptualization.

## Ethical clearance

The study was approved by the Queensland Children's Hospital Human Research Ethics Committee (HREC/25/QCHQ/112471).

## Patient's/ Guardian's consent

As this was a retrospective review of medical records, the requirement for parent or guardian consent was waived.

## Funding

This research did not receive any specific grant from funding agencies in the public, commercial or not-for-profit sectors.

## Declaration of Competing Interest

The authors declare that they have no known competing financial interests or personal relationships that could have appeared to influence the work reported in this paper.
